# Fun with maths: exploring implications of mathematical models for malaria eradication

**DOI:** 10.1186/1475-2875-13-486

**Published:** 2014-12-11

**Authors:** Philip A Eckhoff, Caitlin A Bever, Jaline Gerardin, Edward A Wenger

**Affiliations:** Institute for Disease Modeling, Bellevue, WA USA

## Abstract

Mathematical analyses and modelling have an important role informing malaria eradication strategies. Simple mathematical approaches can answer many questions, but it is important to investigate their assumptions and to test whether simple assumptions affect the results. In this note, four examples demonstrate both the effects of model structures and assumptions and also the benefits of using a diversity of model approaches. These examples include the time to eradication, the impact of vaccine efficacy and coverage, drug programs and the effects of duration of infections and delays to treatment, and the influence of seasonality and migration coupling on disease fadeout. An excessively simple structure can miss key results, but simple mathematical approaches can still achieve key results for eradication strategy and define areas for investigation by more complex models.

## Background

Mathematical models are a helpful tool for testing assumptions and elucidating the quantitative implications of disease features. Most of the time, results of a mathematical modelling study match mental models of expert opinion, which have been shaped by years of research and experience. Occasionally, surprising or counterintuitive results emerge from models. This can happen with simple compartmental models, but sometimes the surprising nature of modelling results is due to mistaken intuition based on mental or simplified mathematical models taken too far. Such findings must be examined closely, but they enhance understanding when validated. This note examines four examples from malaria eradication of how modelling can produce useful results that may initially strike one as unanticipated, especially if one relies too heavily on simple modelling concepts or mental pictures.

## Time to eradication

The classic epidemiological model concept of the basic reproductive number R_0_ nominally represents the secondary infections resulting from a single primary infection in an immunologically naïve population. Macdonald developed the original formulation for malaria [1, 2], and others have extended the theory over time [3]. This concept has become ubiquitous in epidemiological modelling, and while it can be helpful, it can also cause confusion and even misguided planning if used incorrectly. The basic theory is that if R_0_ is reduced to a controlled reproductive number R_c_, the number of secondary infections resulting from a single primary infection under control measures, below 1, then the disease will disappear eventually. Each infection is replaced by less than one infection, and prevalence exponentially decays to zero. While this simplified equilibrium view is true, planning eradication efforts requires investigation of the dynamics and timelines as described by Macdonald and others [4, 5], not just the equilibrium conditions.

**Figure 1:**
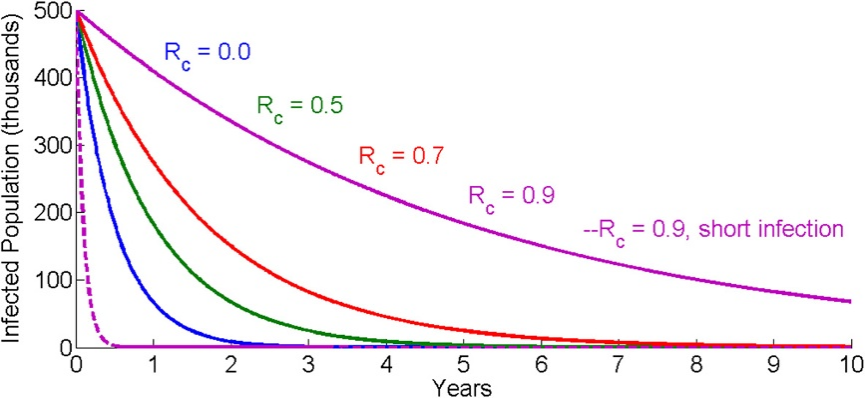
**Decay of the infectious reservoir for different values of R**_**c**_ **, for an average infection duration of 180 days (solid lines) and 3.5 days (dashed line).**

This R_0_ tends to be implemented in simple compartmental models or ordinary differential equations, characterized by an exponential distribution for infection durations. A reduction of R_0_ to R_c_ <1 corresponds to exponential decay of the infected population. The fastest possible decay of the infected population without active clearance of infections is attained when R_c_ = 0 and has the inverse of the average duration of infection as its decay constant. For short duration infections, such as flu, the infectious reservoir decays rapidly. Malaria, on the other hand, has average infection durations of up to six months [6–8]. As such, exponential decay of the infectious reservoir can take years under the best case, and over ten years if R_c_ = 0.9. Figure 1 shows the decay of the malaria infectious reservoir for four values of R_c_, with infection duration averaging 180 days. A controlled reproductive number of 0.9, 0.7, 0.5, or even 0 exhibits slow declines in the total number of infected individuals. In contrast, a disease with R_c_ = 0.9 but an infectious duration of just 3.5 days rapidly depletes its infectious reservoir.

When human infections are not actively cleared and elimination is driven by attaining R_c_ <1 solely through transmission reductions, this slow decay has serious programme implications that limit the chances of elimination. For many diseases the durations of interventions that reduce transmission, such as vaccines, are much longer than the durations of infection. As such, transmission reductions that reduce R_0_ below 1 can succeed in driving local eliminations of the pathogen while the interventions are still efficacious without replacement. For malaria however, the average infection duration of six months is only a factor of six away from three-year lifetimes of insecticide-treated bed nets (ITNs). Indoor residual spraying (IRS) campaigns need to be repeated every two to six months depending on the active ingredient [9] in areas with year-round transmission and need to be repeated yearly in highly seasonal settings. These frequencies are considerably faster than the timelines to elimination based on transmission reductions alone. The current lead vaccine candidate, RTS,S [10], has a duration of efficacy that is not longer than ITNs. As seen in Figure 1, the infectious reservoir may still be quite robust by the time these interventions need to be replaced, or else the reproductive number will rise back above 1 without having achieved elimination.

The result of this matching of decay constants and intervention durability drives elimination programmes down one of two routes. The first is that elimination campaigns need to reduce transmission for many years to maintain R_c_ <1, with repeated distributions. The second possible route is to actively clear the infectious reservoir with drug-based campaigns in order to reduce the duration of infections and to achieve rapid drops in prevalence. Reducing the duration of infections in fact also drives further decreases in R_c_[3].This makes malaria more like diseases of shorter infectious duration and more susceptible to transient transmission-driven reductions of R_c_ <1.

## Coverage and R_0_

In contrast to many common childhood diseases with R_0_ ranging from 1.5 to 12, malaria can exhibit an R_0_ over 100 in well-suited settings [3]. Traditional epidemiological modelling shows that the vaccine coverage required to interrupt transmission with a perfect 100% effective vaccine without decay can be calculated as 1-1/R_0_. The higher the value of R_0_, the higher the covered fraction needs to be. If R_0_ = 2, then exceeding 50% coverage will result in R_c_ <1. If R_0_ = 10, then 90% coverage is required.

**Figure 2:**
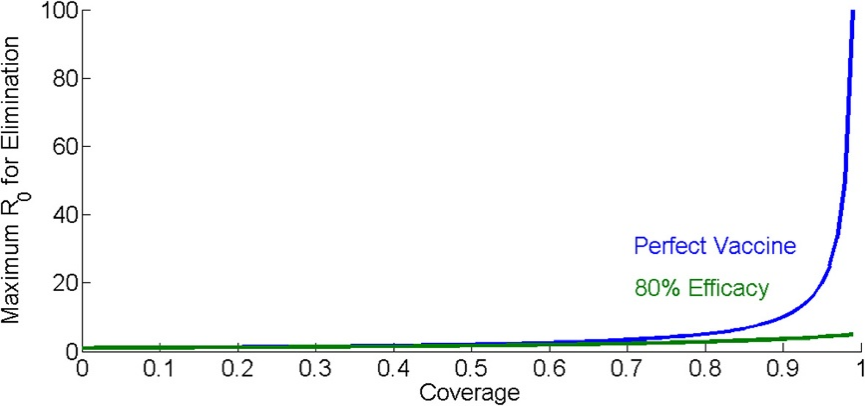
**The maximum R**_**0**_ **that can be eliminated for different levels of vaccination coverage with a perfect infection-preventing vaccine (blue) and one that prevents 80% of infections (green).**

The other side of this relationship is that the higher the coverage, the disproportionately greater R_0_ that can be overcome for each increase in coverage. With a perfect infection-preventing vaccine, one that provides complete sterilizing immunity in vaccinated individuals, high levels of coverage provide dramatic increases in the maximum R_0_ that can be eliminated. Coverage of 50% can eliminate an R_0_ of 2, coverage of 80% can eliminate an R_0_ of 5, and coverage of 90% can eliminate an R_0_ of 10. Above 90%, the gains become dramatic as seen in Figure 2: coverage of 95% will eliminate an R_0_ of 20 and coverage of 99% will eliminate an R_0_ of 100.

This relationship also has implications for the impact of vaccine efficacy. If a vaccine instead of preventing all infections prevents only a fraction V_eff_ of infections, with V_eff_ <1, then the coverage required to achieve R_c_ <1 becomes (1-1/R_0_)/V_eff_. At some combinations of R_0_ and V_eff_, this coverage rises above 100% and elimination becomes impossible even at full coverage. So if the vaccine is leaky and exhibits 80% efficacy at preventing infections, the relationship with maximum R_0_ shifts dramatically at the high end of coverage, as seen in Figure 2. Below 80% coverage, the leaky vaccine can eliminate R_0_’s close to the R_0_’s eliminated by the perfect vaccine. At 90% coverage, however, where the perfect vaccine can eliminate an R_0_ of 10, the leaky vaccine can only eliminate R_0_ up to 4. Even perfect coverage only approaches a maximum R_0_ of 5. The situation becomes even more difficult if vaccine efficacy decays over time, requiring repeated boosting vaccination, higher coverage, higher initial efficacy, or all three.

The result of this relationship is to quantify the value of achieving higher intervention coverage. It is commonly accepted that it becomes increasingly costly to increase coverage at higher baseline coverages. To achieve greater than 90% coverage might be prohibitively expensive. An important counterpoint to these dramatically rising costs of achieving coverage is that one actually buys a much higher eliminate-able R_0_. The high baseline R_0_ exhibited by malaria in some pockets also drives non-linearly increasing value to higher vaccine efficacies. Increasing vaccine efficacies has a challenging set of scientific hurdles and increasing coverage has logistical hurdles, but understanding what is required for success can quantify the value of surmounting either or both sets of hurdles. A similar effect of non-linearly increasing benefits of coverage has been demonstrated in model studies of drug impacts for elimination [11]. In summary, although coverage increases become increasingly expensive as baseline coverage increases, the benefits correspondingly grow.

## Case detection delays and transmission-blocking drugs

There has recently been significant debate about the possible role of low-dose primaquine in elimination scenarios [11]. The basic argument builds from achievable coverage and time constants. If the average duration of a falciparum infection is approximately six months, and the duration of gametocytaemia following clearance with schizonticidal anti-malarial drugs approximately two weeks, then clearing an infection reduces 12 times more onward transmission than adding low-dose primaquine to the schizonticidal regimen. Thus, if MSAT campaigns are reaching 50% of the population with an artemisinin-combination therapy (ACT) to clear infections, adding primaquine to the MSAT regimen for all 50% would only reduce the same amount of transmission as increasing ACT coverage to 54%. Limitations in who could receive primaquine would further reduce this marginal impact. As has previously been pointed out [11], primaquine only begins to have a discernable impact at high coverages, similar to that discussed in the previous section.

However, this calculus depends on the assumption of exponential distributions of infection durations, which is the standard implementation in most compartmental and ordinary differential equation (ODE) based models [12]. Since an exponential distribution is memoryless, no matter how long an infection has lasted, its expected remaining duration remains the average duration of an infection. So in a compartmental or ODE-based model, any cleared infection removes six months of residual transmission. As has been seen [6–8], durations of falciparum infections are not exponentially distributed. In primary infections in tertiary syphilis patients, very few infections were cleared before three months, although the average duration was still in the order of half a year.

**Figure 3:**
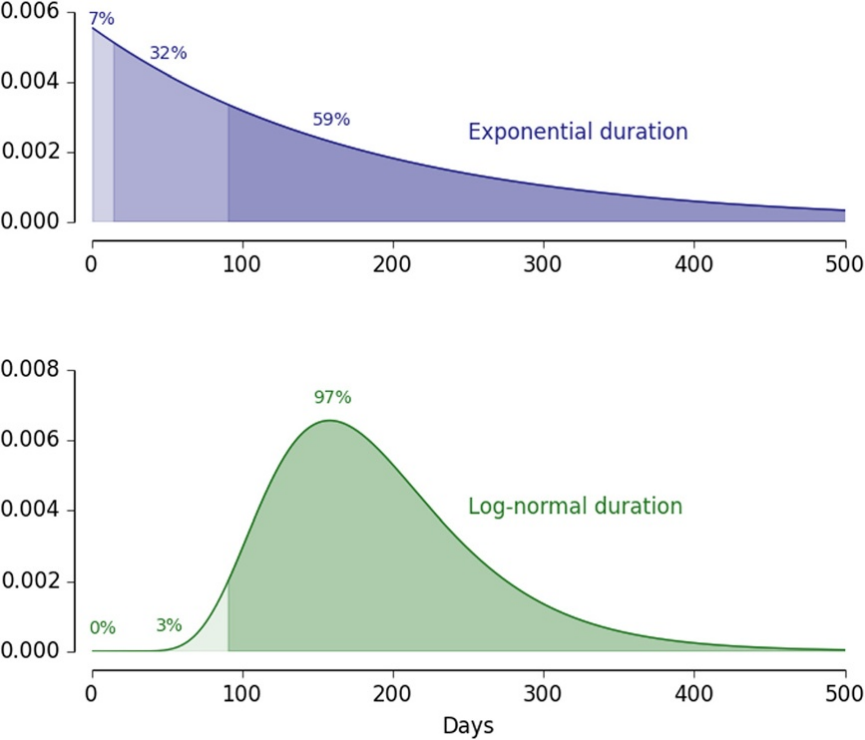
**The difference in remaining distribution of infection duration following a 90-day case detection lag for an exponential distribution (blue) and a log-normal distribution (green), each with a mean of 180 days.**

With a non-exponential distribution of infection durations, the calculated relative impact of primaquine can change. Suppose a three-month delay from infection onset to treatment: under the simple exponential assumption, clearing that infection prevents six months of transmission on average. Under the more realistic distribution, which is not memoryless, the three-month delay in case finding matters. For infections that have lasted three months, the expected remaining duration is now only three months instead of six months, as few infections have been cleared by 90 days. Figure 3 shows the difference in the probability of infections lasting less than 90 days for an exponential distribution and a log-normal distribution, both with the same mean duration. However, the two-week reduction in transmission due to primaquine remains two weeks, now just a six-fold smaller relative impact. The longer the delay in case finding, the higher is the relative impact of reducing onward transmission of adding primaquine to the schizonticidal regimen compared to just treating with a schizonticidal regimen.

For drug-based clearance of the infectious reservoir, the most important factors remain achieving high coverage with ACT and reducing the delay in case finding, but the impacts of realistic delays in case finding and data-driven distributions of infection duration constitute an important, although minor, effect. This is an example in which excessively simple assumptions may get the basic result correct but miss an important nuance, while somewhat more detailed mathematical approaches can capture realistic effects and improve accuracy. Realistic delays in case finding and data-driven distributions of infection duration together reduce the target ACT coverage at which adding a transmission blocking drug (not necessarily primaquine) to the ACT regimen begins to show equivalent epidemiological impact to a fixed fraction increase in coverage with the schizonticidal regimen alone. Compared to the previous two examples, this impact of infection senescence is a minor effect. However, this effect can still be elucidated with mathematical analyses.

## Metapopulations and synchrony

While malaria transmission is very much a local phenomenon driven by mixing of mosquitoes and humans on short spatial scales, malaria eradication is a global issue. Areas are connected spatially by human migration and elimination is not possible without considering the spatial context. Excellent work has been done to show the impact of human mobility on malaria [13], and how the coupling of regions raises the difficulty of achieving elimination. Human movements superimposed on a landscape of heterogeneous transmission intensities and campaign coverages have the potential to reintroduce infection to temporarily cleared areas before the benefits of elimination ‘stickiness’ can be achieved, contributing to malaria resurgence [14, 15]. As such, initiating an elimination campaign is largely a regional, rather than a local decision [16].

**Figure 4:**
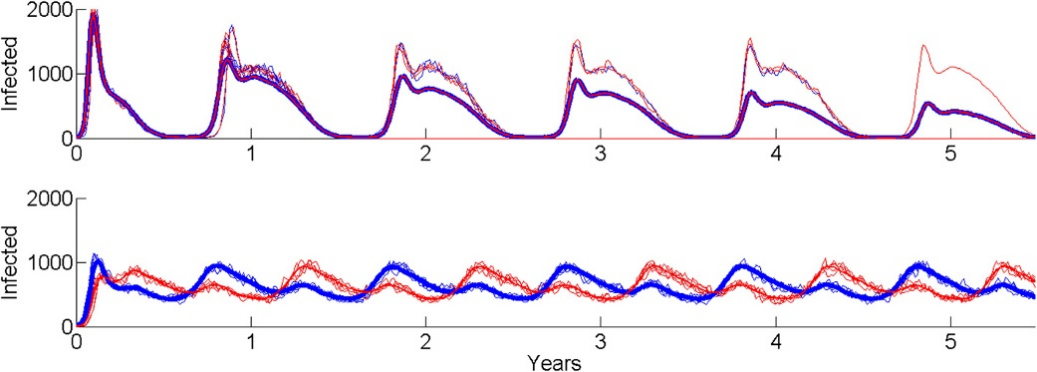
**Two locations (red and blue) linked by migration with synchronous (top) and asynchronous (bottom) forcing.** The mean number of infected individuals over 100 runs is plotted in bold for each location, along with representative trajectories. No fade-out occurs in the asynchronous simulation, and the ensemble is continuously depleted by fade-out in the synchronous case. The peak number of cases is substantially higher in the synchronous case, however.

Within this spatial context, however, mathematical modelling can help in planning and understanding how spatial transmission dynamics affect disease fade-out. Herein lies an easily overlooked result. In a spatially structured population with local transmission among subpopulations and migration from one subpopulation to another, these metapopulation dynamics affect whether there is disease fade-out over the whole population. If there are oscillations, perhaps due to seasonality, then research has shown that synchrony among the metapopulations actually increases the chance of fade-out [17]. If the oscillations in disease prevalence are synchronous, then all pockets go through the low point at about the same time. This increases the probability of all simultaneously fading out, which is the definition of elimination. The result is that more synchronous behaviour actually helps elimination, even if it coincides with higher peak case counts. A temporally stable transmission pattern is actually more difficult to extinguish, even if the peak season is not as dramatically bad. This phenomenon is illustrated in Figure 4, which implements two coupled populations with stochastic susceptible-infected-recovered-susceptible (SIRS) dynamics and synchronous (top) and asynchronous (bottom) forcing, with homogeneous mixing within each population. Even though the peak number of cases is higher in the synchronous case, the coupled system exhibits stochastic fade-out each low season, as seen in the mean of 100 runs of the system trending downwards over time. The asynchronously forced system, which having less dramatically bad high seasons, never comes close to extinction.

This benefit of synchrony for achieving elimination has several implications for malaria eradication. The campaign benefits from seasonal forcing being similar in phase over locally connected areas, and the chance of elimination increases if anti-malarial treatments are used to shorten the length of infections sufficiently to have rapid decays of the infectious reservoir during the low transmission season. In addition, locations with more stable year-round transmission, even if they do not have the high spikes in case counts of highly seasonal settings, will be more difficult to eliminate and may require higher campaign coverages for a given annual average R_0_. Finally, achieving elimination across connected geographic regions will be easier if the external forcing on transmission, namely campaign pressures such as vector control spraying and anti-malarial drug mass screen-and-treating, are synchronized across the region of interest.

## Conclusions

In summary, each of these four examples demonstrates the power of data-driven mathematical analyses for informing malaria eradication strategy. Mathematical modelling forces one to make assumptions explicit and reveals the implications of those assumptions. These examples also show how model structure can impact results and conclusions for policy while illustrating the importance of comparing results from simple and complex models. Dynamics and small populations matter in an eradication context, so checking equilibrium analyses with both stochastic and deterministic dynamic models is essential.

Each of the four examples in this note can be examined in the relatively simple frameworks here or in more complex microsimulation models. Simple mathematical analyses can often arrive at the correct big picture, while small modifications can capture nuances that improve accuracy, as in the third example. More complicated models can then confirm results while investigating whether the effects of realistic details affect policy conclusions or not. Since it would be possible to investigate irrelevant details in complex models forever, simple models have an additional important role in focusing attention on areas where the capacities of complex models could influence the answer. Starting with a simple framework is thus strategically useful.

It is important to make sure that seemingly straightforward conclusions from a simple model structure be rigorously examined with model structures that can accommodate improved realism [11, 18–20]. If the same result for a given question is obtained from simple and complex models, then one gains confidence in the results from the simpler structures. More complex microsimulation models can recreate and test results obtained in simple analyses and facilitate extensions of questions that would have been otherwise less tractable. Such questions raised by the examples above could include the interrelationships among decay of vaccine efficacy, heterogeneity in vaccine coverage, delivery strategy and schedule, transmission context, and coverage targets or the effects on disease fadeout of spatial variation in seasonality and local heterogeneity in risk. When used properly in concert, a diversity of mathematical models and approaches will play an essential role in eradication planning.
